# *LITAF* is a potential tumor suppressor in pancreatic cancer

**DOI:** 10.18632/oncotarget.23220

**Published:** 2017-12-14

**Authors:** Yuan Zhou, Jing Huang, Xi Yu, Xin Jiang, Yaoyao Shi, Yuanyuan Weng, Yue Kuai, Lizhen Lei, Guoping Ren, Xiaowen Feng, Guoping Zhong, Qingmeng Liu, Hongyang Pan, Xinxia Zhang, Ren Zhou, Caide Lu

**Affiliations:** ^1^ Department of Hepatopancreatobiliary Surgery, Ningbo Medical Center Lihuili Eastern Hospital, Ningbo, China; ^2^ Medical School of Ningbo University, Ningbo, China; ^3^ Department of Pathology and Pathophysiology, Institute of Pathology and Forensic Medicine, Zhejiang University School of Medicine, Hangzhou, China; ^4^ The First Affiliated Hospital, Zhejiang University School of Medicine, Hangzhou, China; ^5^ Division of Pathology, Yinzhou Hospital Affiliated to Medical School of Ningbo University, Ningbo, China; ^6^ Division of Pathology, The Second People’s Hospital, Shaoxing, China; ^7^ Epitomics (Hangzhou) Inc., Hangzhou, Zhejiang, P.R. China

**Keywords:** lipopolysaccharide-induced tumor necrosis factor-α factor (LITAF), pancreatic cancer, promoter methylation, tumor suppressor gene

## Abstract

Early diagnosis of pancreatic cancer, one of the most deadly cancers with low survival rates, is difficult, and effective biomarkers are urgently needed. Lipopolysaccharide-induced tumor necrosis factor-α factor (*LITAF*) has been recently proposed as a potential tumor suppressor gene in several types of cancer. Here, we analyzed the biological function of *LITAF* in pancreatic cancer. The *LITAF* gene and protein levels were decreased in pancreatic tumor tissues compared with their paired adjacent non-cancerous tissues. In addition, patients with the lower *LITAF* protein expression had lower disease-free survival rates. The decreased *LITAF* expression correlated with *LITAF* promoter hypermethylation in pancreatic cancer cells and tissues. Moreover, promoter demethylation dose-dependently increased the *LITAF* transcription. Importantly, *LITAF* demethylation suppressed proliferation and cell cycle progression, and enhanced apoptosis of pancreatic cancer cells. Together, our results indicate that *LITAF* functions as a tumor suppressor gene in pancreatic cancer cells, and might serve as a novel biomarker for early diagnosis of pancreatic cancer.

## INTRODUCTION

Pancreatic cancer is a leading cause of cancer-related death with an overall 5-years rate of less than 5% [1]. Although some progress has been made in therapy of pancreatic cancer, there have been few improvements in overall survival (OS), largely because of the difficulty of early diagnosis [2, 3]. Most patients are diagnosed with advanced stage disease and the median survival with therapy is less than 6 months [4, 5]. Even for the minority of patients undergoing surgery, the 5-years OS is only 20% after resection [6]. Early detection of pancreatic cancer has been identified as the best way to improve patient survival [7–10]. However, there is currently no reliable and noninvasive screening test for this cancer.

Several genes have been identified as promising targets in diagnosis and therapy of pancreatic cancer. For instance, *KRAS, p16/CDKN2A, TP53 and SMAD4* are altered in >50% of pancreatic cancer cases [11]. Mutations of *KRAS* might be used to detect pancreatic cancer in early stage [12] and *SMAD4* mutations associate with dismal prognosis of pancreatic neoplasia [13, 14]. Similarly as *p16/CDKN2A* and *TP53* tumor suppressors, lipopolysaccharide-induced tumor necrosis factor-α factor (*LITAF*) has been considered as a tumor suppressor gene because its expression is regulated by *P53* [15]. Several cohort studies have indicated that *LITAF* is abnormally expressed in cancerous tissues compared with normal tissues [16–19]. Nevertheless, the mechanism of its aberrant expression is still unknown. Matsumura *et al.* reported that somatic mutations in the *LITAF* gene are associated with its aberrant expression in extramammary Paget’s disease (EMPD) samples [15]. However, the relationship between *LITAF* somatic mutations and its aberrant expression is unclear, and the *LITAF* function in pancreatic cancer is unknown.

In this study, we investigated the *LITAF* function in pancreatic cancer. In addition, we analyzed the methylation status of the *LITAF* promoter in pancreatic cancer cells and tissues. Our results indicate that LITAF functions as a tumor suppressor in pancreatic cancer, and may serve as potential biomarker for early diagnosis of pancreatic cancer.

## RESULTS

### Expression of *LITAF* is downregulated in most cases of pancreatic cancer

**Figure 1:**
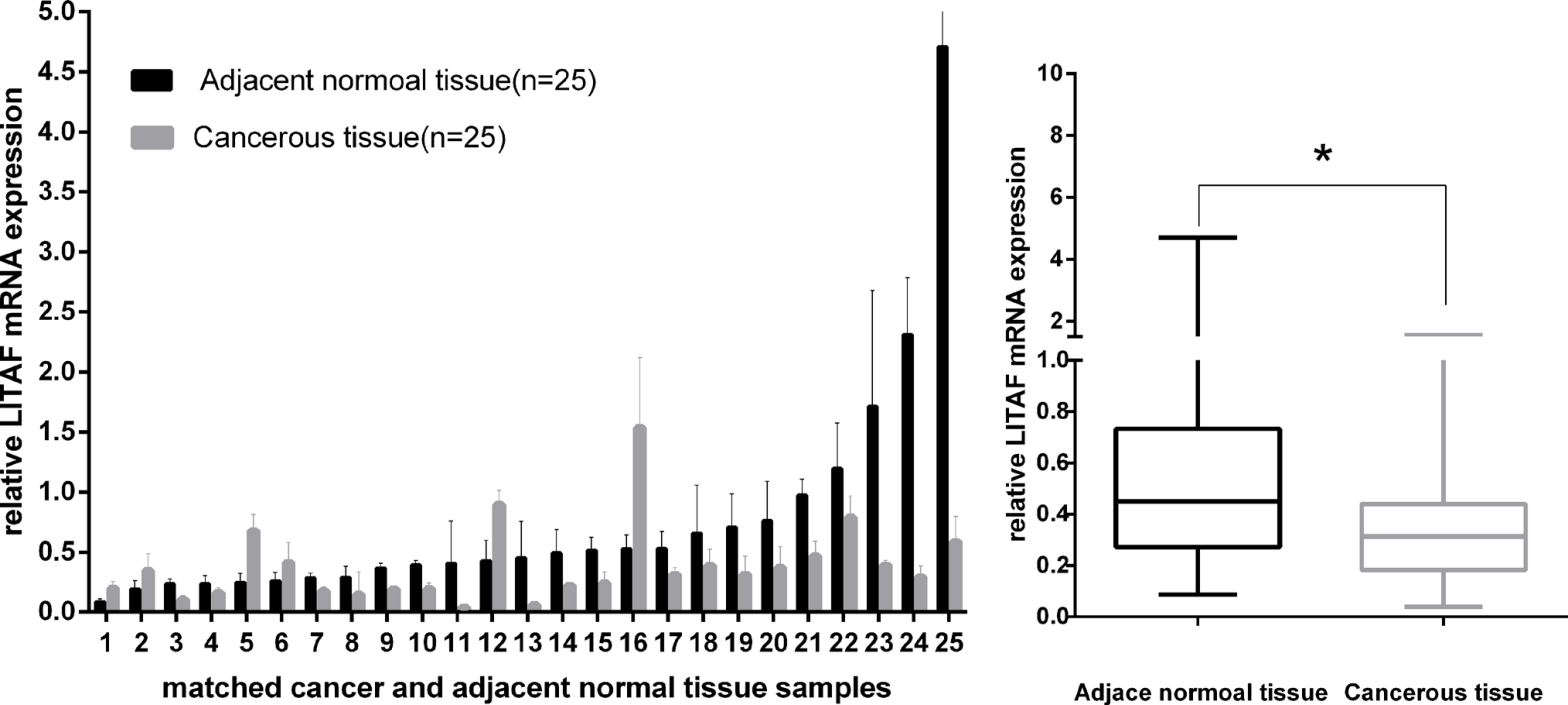
*LITAF* gene expression in 25 paired pancreatic tumor tissues *LITAF* mRNA expression was decreased in most tumor cases (19/25, 76%) compared with adjacent normal tissues. Box plot analysis illustrated the reduced *LITAF* mRNA expression in tumor tissues with an average expression level of 0.3804 compared with adjacent normal tissues (mean expression level, 0.7573) ^*^p < 0.05, However, mRNA expression of *LITAF* was increased in 6 cases (6/25, 24%).

The mRNA expression of *LITAF* was evaluated in fresh tissues of 25 pancreatic cancer cases by using RT-qPCR. As shown in Figure 1, *LITAF* mRNA levels were downregulated in 19 out of the 25 cases (76%) compared with their adjacent non-tumor tissues (*p* < 0.05). Interestingly however, we found that *LITAF* mRNA expression of 6 cases (24%, cases 1, 2, 5, 6, 12 and 16) was actually upregulated compared with their adjacent non-tumor tissues. From these 6 cases, cases 1, 2, 5, 6, and 16 were all ductal adenocarcinoma, and case 12 was neuroendocrine carcinoma.

**Figure 2:**
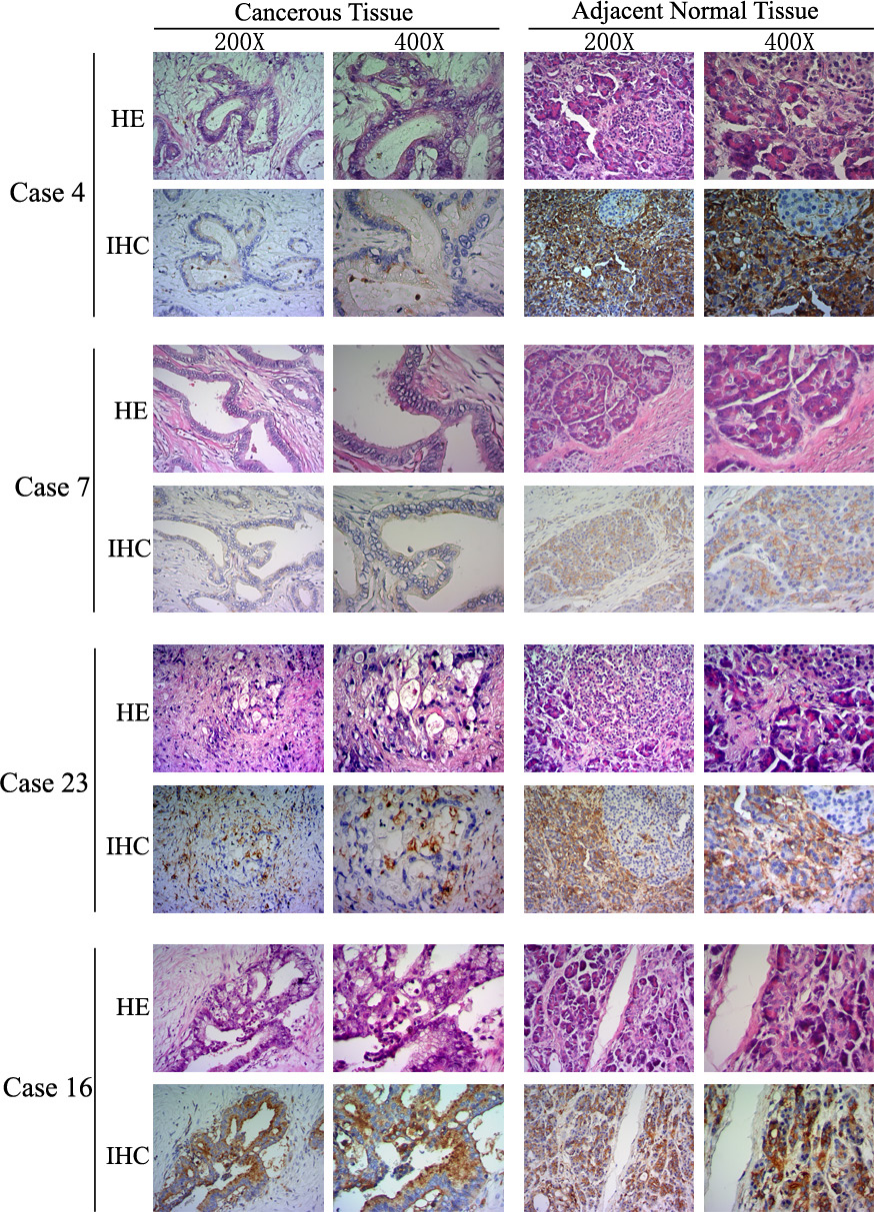
LITAF protein expression in human pancreatic cancer LITAF proteins levels were analyzed by IHC in 50 paired pancreatic cancer tissues. Figure 2 illustrates examples using cases with absent/weak LITAF immunostaining (4, 7, and 23), and intense immunostaining (case 16). The histopathological morphology and IHC images are presented using × 200 and × 400 objective magnifications, respectively.

The expression of *LITAF* in the pancreatic tumors was further confirmed at protein level by using immunohistochemical (IHC) staining. We examined *LITAF* protein expression in 25 paired fresh tumor tissues (as mentioned in Figure 1), and 25 paired paraffin-embedded tumor tissues. IHC results showed that 21 out of the total 50 cases (42%) had absent/weak cytoplasmic LITAF levels, whereas 29 tumor tissues (58%) showed strong cytoplasmic staining (Figure 2). The findings suggest that pancreatic cancer can be divided into two subtypes, according to the LITAF expression.

In order to investigate the relationship between clinical characteristics and *LITAF* expression, we classified the data sets by clinical-pathologic characteristics (i.e., age, gender, tumor size, TNM stage, lymph nodes involvement) and *LITAF* expression status. The clinical-pathologic characteristics of patients are summarized in Table 1; the statistical analysis results are shown in Table 2. There was no significant difference between expression of *LITAF* detected by IHC or RT-qPCR and clinical-pathologic characteristics. However, the study on clinical characteristics included 50 pancreatic tumors including 40 ductal adenocarcinomas and 10 other types, such as neuroendocrine neoplasms and solid pseudopapillary neoplasm. Since different types of pancreatic tumors have different biological behavior, we re-analyzed the relationship between clinical characteristics and *LITAF* expression in 40 ductal adenocarcinomas and achieved similar results (Supplementary Tables 1–2).

**Table 1:** Clinicopathologic characteristics of patients included in this study

Characteristic	Case (%)	Median (Range)
**Age (years)**		
≥65	23 (46.0)	62 (24,82)
<65	27 (54.0)	
**Gender**		
male	26 (52.0)	
female	24 (48.0)	
**Tumor Size (cm)**		
≤2	9 (18.0)	
>2	41 (82.0)	
**Lymph node involvement**		
positive	24 (48.0)	
negative	26 (52.0)	
**TNM Stage (WHO Classification)**		
Ia	2 (4.0)	
Ib	15 (30.0)	
IIa	8 (16.0)	
IIb	22 (44.0)	
III/IV	3 (6.0)	
**Pathology**		
Ductal adenocarcinoma	40 (80.0)	
Neuroendocrine neoplasms	6 (12.0)	
Intraductal papillary mucinous carcinoma	2 (4.0)	
Solid-pseudopapillary neoplasm	1 (2.0)	
Mucinous cystic carcinoma	1 (2.0)	

**Table 2:** Correlation between clinical characteristics and *LITAF* expression

	LITAF mRNA Expression (Q-PCR)				LITAF protein Expression (IHC)			
	High	Low	*N*	*p*	High	Low	*N*	*p*
**Age (years)**								
≥65	0	10	25	0.051	12	10	50	0.662
<65	6	9			17	11		
**Gender**								
male	4	8	25	0.378	15	11	50	0.963
female	2	11			14	10		
**Tumor Size (cm)**								
≤2	2	1	25	1.0	3	6	50	0.20
>2	5	17			26	15		
**Lymph Nodes**								
positive	1	10	25	0.180	16	11	50	0.865
negative	5	9			13	10		
**TNM Stage**								
I/II	4	19	25	0.05	27	20	50	1.0
III/IV	2	0			2	1		

### *LITAF* expression correlates with survival in pancreatic cancer patients

**Figure 3:**
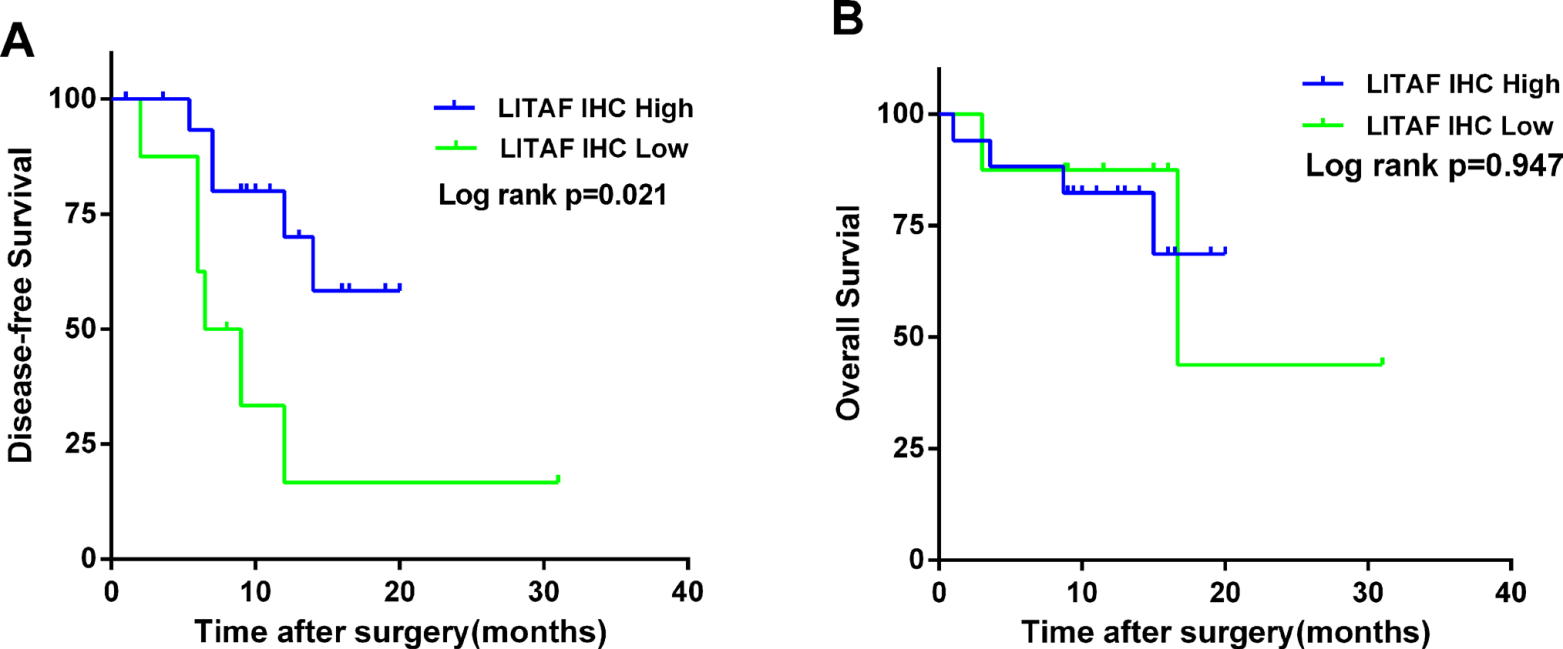
LITAF protein expression correlates with survival in patients with pancreatic cancer The Kaplan–Meier (KM) method was used to analyze DFS and OS according to the LITAF protein expression. (**A**) The Kaplan–Meier curve for DFS revealed a poorer DFS in patients with low LITAF expression (median 7.25 months vs 11 months, Log rank *p* = 0.021). (**B**) The Kaplan–Meier curve for OS demonstrated that there was no significant difference in OS according to LITAF expression (median 13.25 vs 12.5 months, Log rank *p* = 0.947). Green line, low LITAF expression. Blue line, high LITAF expression.

Based on the IHC results, we investigated the relationship between patient survival and LITAF expression in 25 patients. 8 patients had a low LITAF expression (IRS ≤ 1), while17 patients had a high LITAF expression (IRS ≥ 9). Surprisingly, patients with the low LITAF expression had a significantly poorer DFS (*p* = 0.021, Figure 3A). There was no significant association between LITAF expression and OS (*p* = 0.947, Figure 3B). However, the patients involved in survival analysis had different pancreatic tumors types, including 20 ductal adenocarcinomas, 2 neuroendocrine neoplasms, 2 intraductal papillary mucinous carcinomas and 1 solid-pseudopapillary neoplasm. Similar results were observed as we re-analyzed OS and DFS of 20 ductal adenocarcinomas (Supplementary Figure 1).

### *LITAF* transcription is regulated by promoter methylation in pancreatic cancer cells and tissues

**Figure 4:**
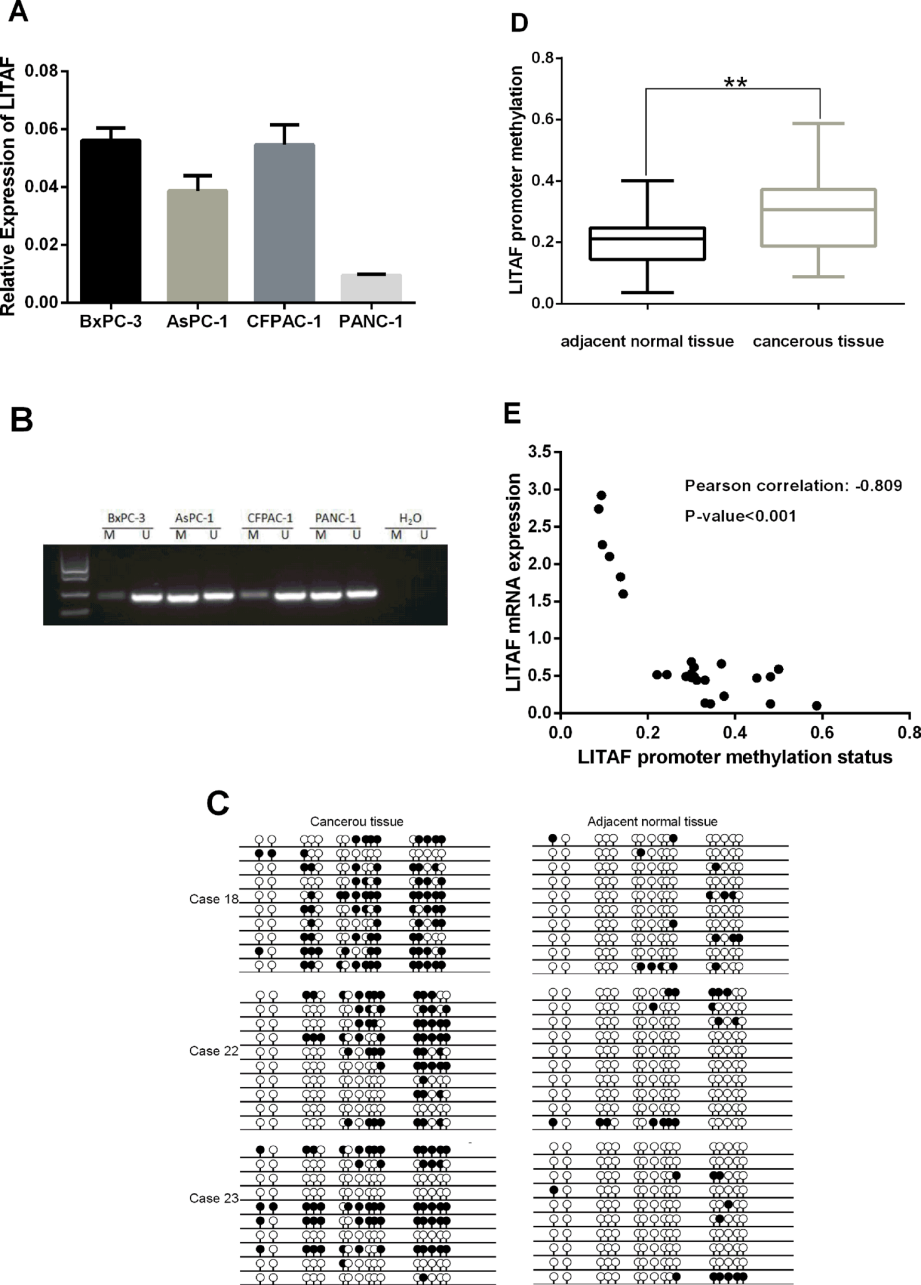
Transcription of *LITAF* is regulated by promoter methylation (**A**) The *LITAF* mRNA expression was examined by RT-qPCR in four pancreatic cancer cell lines. The downregulated *LITAF* was detected in AsPC-1 and PANC-1 cells, which showed intense methylation (shown in Figure 4B), whereas high *LITAF* expression was detected in BxPC-3 and CFPAC-1 cells with weak methylation (Figure 4B). Triplicate tests for each cell line, X ± SD. (**B**) Analysis of the methylation status of the *LITAF* promoter region covering a 240 bp fragment in BxPC-3, AsPC-1, CFPAC-1, and PANC-1 cells. A visible PCR product in lane-U indicates the presence of unmethylated promoters; the presence of product in lane-M indicates the presence of promoter methylation. Intense methylation was detected in AsPC-1 and PANC-1 cells, and weak methylation was detected in BxPC-3 and CFPAC-1 cells. (**C**) Methylation status of *LITAF* promoter covering a 240 bp fragment with 16 CpG sites in paired pancreatic neoplasm samples was confirmed by BSP. Each row represents one bacterial clone with one circle symbolizing one CpG site. 10–15 clones were randomly selected and sequenced for each sample. Filled circles (●) indicated the methylated. Open circles (○) indicated the unmethylated. BSP analysis revealed the methylation on *LITAF* promoter was significantly increased compared with adjacent normal pancreatic tissue. (**D**) Box plot illustrates significant *LITAF* promoter hypermethylation in 25 paired fresh cancerous tissues compared with adjacent normal tissues (mean methylation level: 20.22% vs 30.03%, ^**^p < 0.01). (**E**) Scatter plot showed the inverse association between *LITAF* mRNA expression and DNA methylation status of the *LITAF* gene in 25 pancreatic tumor tissues. Pearson correlation: *r* = –0.809, *p* < 0.001.

To understand the molecular mechanisms regulating *LITAF* transcription in pancreatic cancer, we first analyzed *LITAF* mRNA expression in four pancreatic cancer cell lines (BxPC-3, AsPC-1, CFPAC-1 and PANC-1) by RT-qPCR. *LITAF* expression was higher in BxPC-3 and CFPAC-1 cells, and lower in AsPC-1 and PANC-1 cells (Figure 4A). To investigate if promoter methylation might be responsible for the downregulation of *LITAF*, we examined the methylation status of the promoter region of *LITAF* by using MSP and BSP in the four pancreatic carcinoma cell lines and 25 paired pancreatic cancer samples. Intense methylation was detected in AsPC-1 and PANC-1 cells that had the low *LITAF* expression, whereas BxPC-3 and CFPAC-1 cells with the high *LITAF* expression showed low methylation (Figure 4B). Additionally, there was a significant inverse association between *LITAF* mRNA expression and methylation status of *LITAF* promoter in 25 paired pancreatic tumor tissues (*p* < 0.001, Figure 4E).

Next, we analyzed the methylation levels of *LITAF* promoter in tumor samples and adjacent normal tissues. The BSP analysis covered a 240bp-DNA fragment with 16 CpG sites on *LITAF* promoter and revealed the methylation status on *LITAF* promoter (Figure 4C). The methylation levels of *LITAF* promoter were significantly increased in 25 pancreatic cancer tissues compared with adjacent normal tissues (*p* < 0.01, Figure 4D); 3 typical pancreas cancer cases (case 18, 22 and 23) with hypermethylation in *LITAF* promoter are shown in Figure 4C.

### Methylation of *LITAF* promoter decreases *LITAF* expression in pancreatic carcinoma cells

**Figure 5:**
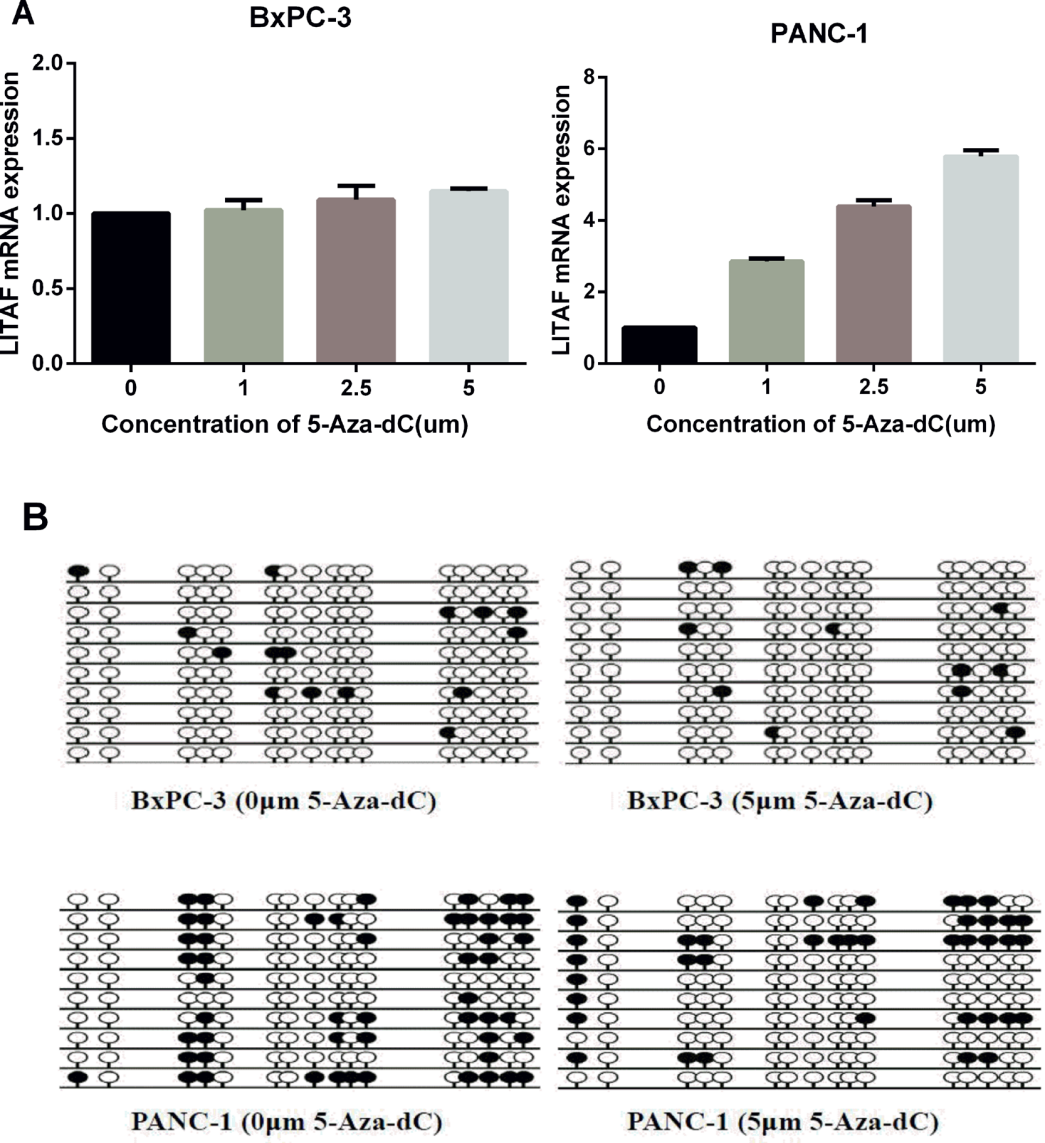
*LITAF* promoter methylation regulates *LITAF* expression in pancreatic carcinoma cells (**A**) PANC-1 and BxPC-3 cells were treated with 0, 1, 2.5 or 5 μM 5-Aza-dC for 72 h, and *LITAF* mRNA levels were determined by RT-qPCR. The results represent triplicate tests for each concentration point, X ± SD. (**B**) The methylation status of *LITAF* promoter covering a 240 bp region with 16 CpG sites measured in cells treated with and without 5-Aza-dC (5 μM). Each row represents one bacterial clone with one circle symbolizing one CpG site. 10–15 clones were randomly selected and sequenced for each sample. Filled circles (●) indicated the methylated sites; open circles (○) indicated the unmethylated sites.

Based on the above data, we further analyzed the impact of promoter methylation status on *LITAF* mRNA expression in pancreatic cancer cells. Both PANC-1 cells (with intense promoter methylation and weak *LITAF* expression) and BxPC-3 cells (with weak promoter methylation and high *LITAF* expression) were treated with the demethylation agent 5-Aza-dC for 72 h at different dosages of 0, 1, 2.5 and 5 μM. The *LITAF* levels in PANC-1 cells were dose-dependently upregulated at 5-Aza-dC, while there was no significant difference in *LITAF* expression in BxPC-3 cells (Figure 5A).

BSP analysis of the DNA methylation status showed that the methylation level of *LITAF* promoter decreased in PANC-1 cells after treatment with 5-Aza-dC, while it did not change in BxPC-3 cells (Figure 5B). These data indicate that pancreatic carcinoma cells with intense promoter methylation are sensitive to demethylation by 5-Aza-dC, and suggest that the decreased methylation of *LITAF* promoter contributes to the upregulation of *LITAF* mRNA.

### *LITAF* demethylation inhibits cell growth, and induces apoptosis and cell cycle arrest in pancreatic carcinoma cells

**Figure 6:**
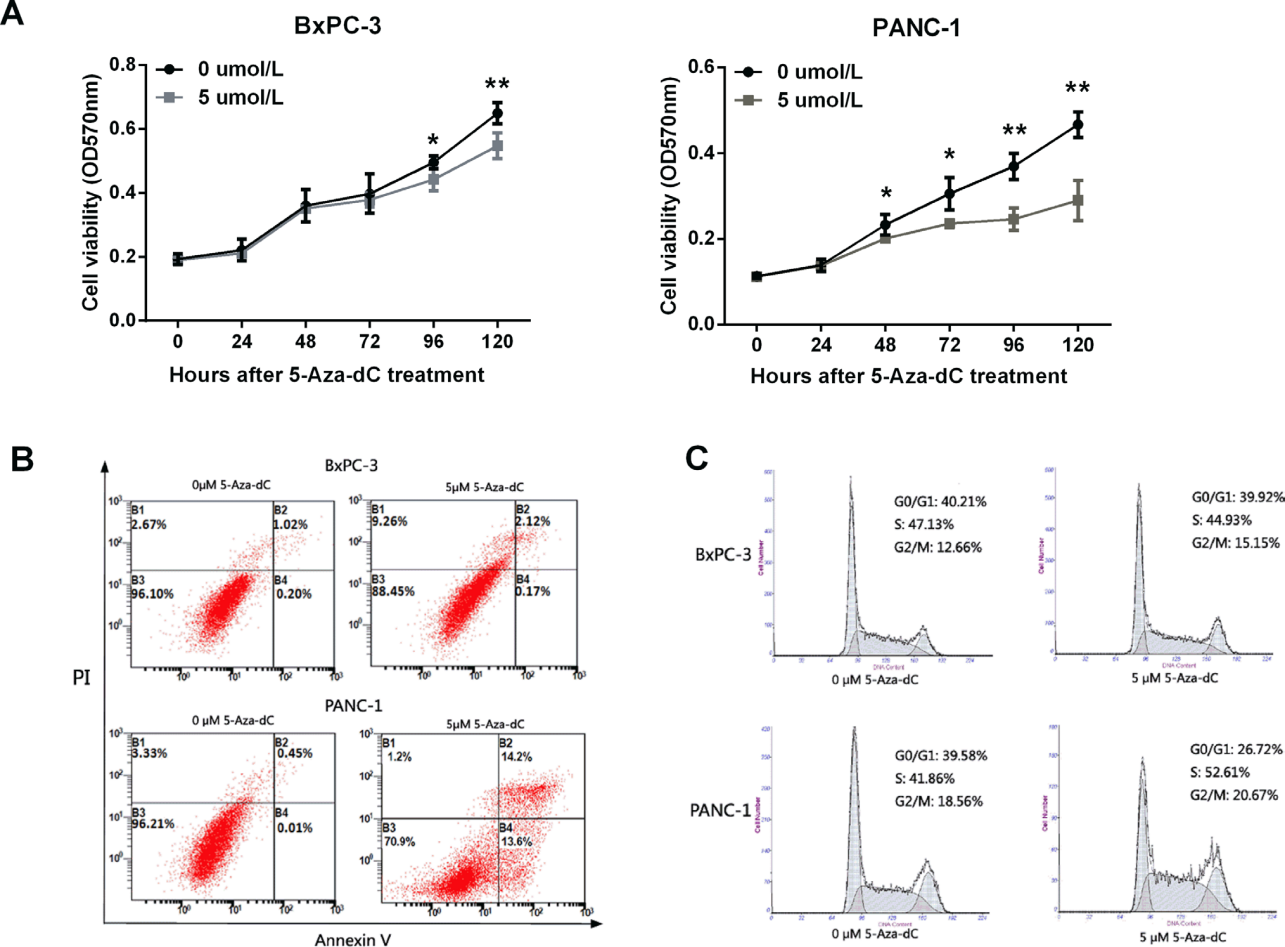
LITAF demethylation inhibits cell growth, and induces apoptosis and cell cycle arrest in pancreatic carcinoma cells (**A**) Growth of BxPC-3 and PANC-1 cells treated with 5-Aza-dC (0 to 5 μM) was analyzed using MTT assay. The results represent four measurements for each time point; X ± SD; ^*^p < 0.05, ^**^p < 0.01. (**B**) Flow cytometry apoptosis assay with PI and Annexin V staining. (**C**) Flow cytometry analysis of cell cycle measured in cells treated with 5 μM 5-Aza-dC for 72 h.

In order to evaluate the *LITAF* function in regulating tumorigenesis of pancreatic cancer cells, cell proliferation, apoptosis, and cell cycle were analyzed. The cell growth of PANC-1 cells was suppressed after treatment with 5 μM 5-Aza-dC (Figure 6A, ^*^p < 0.05, ^**^p < 0.01). However, an inhibition of cell growth was observed also in BxPC-3 cells (Figure 6A, ^*^p < 0.05, ^**^p < 0.01). To investigate whether this cell growth suppression was caused by apoptosis, PANC-1 and BxPC-3 cells were stained with propidium iodide (PI) and Annexin V after treatment with 5-Aza-dC for 72 h, and apoptosis was assessed by flow cytometry. The apoptosis rate of PANC-1 cells treated with 5 μM 5-Aza-dC was 27.8%, much higher than control cells (Figure 6B). However, there was no significant difference in the apoptosis rates of BxPC-3 cells after treatment with 0 μM and 5 μM 5-Aza-dC.

To evaluate whether cell cycle could be impacted after demethylation of *LITAF*, flow cytometry analysis was performed in PANC-1 and BxPC-3 cells. The analysis revealed a significant increase in the number of cells in the S phase in 5-Aza-dC treated PANC-1 cells, but not BxPC-3 cells (Figure 6C). The number of PANC-1 cells in G0/G1 phase decreased remarkably, while G2/M phase did not change (Figure 6C). Together, these results indicated that *LITAF* demethylation induces LITAF expression and cell cycle block at the S phase checkpoint in pancreas carcinoma cells

## DISCUSSION

Recent studies have shown that *LITAF* is frequently down-regulated in different types of cancer [20], including acute leukemia [17], breast cancer [16, 21], lymphoma [22, 23], and prostatic cancer [24], suggesting that it may function as a tumor suppressor gene. The downregulation of LITAF induces migration, increased cell viability, and colony formation in cancer cells [25].

Most pancreatic cancers are unresectable at diagnosis, with an overall 5-year survival rate of less than 5% [1]; the resectable surgery can be performed only in 15% of candidate patients [26]. Early detection is crucial to improve the survival rates of pancreatic cancer patients; yet, reliable and non-invasive biomarker are missing. In this study, we have investigated the *LITAF* function in pancreatic cancer to test whether it could serve as a potential biomarkers for early diagnosis and target therapy.

Similarly to studies in other solid cancers, we found that the expression of *LITAF* was downregulated in most of pancreatic cancer cases (76%, 19/25); most of them were pancreatic ductal adenocarcinoma. Interestingly however, we found that the expression of *LITAF* mRNA was actually increased in 6 pancreatic cancer cases (24%, 6/25) compared with their paired non-tumor tissues (Figure 1). Among the 6 cases, 5 cases were pancreatic ductal adenocarcinomas, and one case was neuroendocrine carcinoma. This might suggest that pancreatic ductal adenocarcinoma might be further divided into at least two subgroups: with and without high *LITAF* expression. A similar result was also observed by analyzing the LITAF protein expression. In a large set of 50 cases including 25 paired fresh tumors and 25 paired paraffin-embedded tumors, 42% (21/50) cases showed absent/weak immunostaining, whereas 58% (29/50) cases showed intense LITAF immunostaining (Figure 2). The immunostaining results also indicated two different LITAF expression patterns in pancreatic cancer. Future studies should confirm these observations using a large number of pancreatic cancer cases.

Interestingly, low expression of *LITAF* correlated with a poorer DFS (Figure 3), although there was no significant difference on OS. These results suggest that *LITAF* might be used as a potential prognosis marker in pancreatic cancer patient; however, future studies should use longer follow-up periods to confirm these data.

To study the tumorigenesis mechanisms of pancreatic cancer, most previous studies have focused on gene mutations, transcriptional activation, and signal transduction [11–14, 24, 27]. Here, we investigated the biological function of *LITAF* through the epigenetic analysis of pancreatic cancer cells and tissues. Our results demonstrate that the methylation degree of *LITAF* promoter regulates the *LITAF* expression (Figures 4 and 5), suggest that the *LITAF* promoter methylation may represent one of the mechanisms responsible for the pathogenesis of pancreatic cancer. Our epigenetic data show that demethylation of the *LITAF* promoter inhibits cell proliferation, survival and cell cycle of pancreatic carcinoma cells. Our results suggest that the methylation status of the *LITAF* promoter may be considered as a candidate biomarker for molecular therapy of pancreatic cancer. Whether *LITAF* might serve as a target gene in early diagnosis of pancreatic cancer needs further analysis in patients with early-stage pancreatic cancer.

In addition, in our epigenetic study of the *LITAF* promoter in pancreatic cancer cells and tissues, we noticed not all of pancreatic cancer cases and cells had the same reaction to aberrant methylation of *LITAF* promoter, indicating existence of different molecular subtypes.

Together, our results indicate that LITAF functions as a tumor suppressor in pancreatic cancer cells. More studies will be needed to determine whether *LITAF* might serve as a pancreatic cancer biomarker, and whether it might be used stratify different molecular subtypes of pancreatic cancer.

## MATERIALS AND METHODS

### Patients and tissue samples

All human primary pancreatic neoplasm tissues and adjacent nonmalignant tissues were obtained from Hepatobiliary & Pancreatic Surgery Department of Ningbo Medical Treatment Center Lihuili Eastern Hospital and Pathology Center in Ningbo. Specimens were stored at −80°C for molecular analysis. Pathological diagnosis was done and confirmed by at least two senior pathologists at the Pathological Center in Ningbo. The histopathology of tumors was classified by AJCC classification. This study was approved by the Ethics Committee of Ningbo University (Ningbo, China).

### Cell culture

Four pancreatic carcinoma cell lines (BxPC-3, AsPC-1, CFPAC-1, and PANC-1) were provided by Department of Pathology and Pathophysiology, Institute of Pathology and Forensic Medicine, Zhejiang University School of Medicine. All cell lines were maintained in RPMI 1640, DMEM or IMDM medium supplemented with 10% fetal bovine serum (GIBCO Invitrogen, USA) in a humidified incubator at 37°C with an atmosphere of 5% CO_2_.

### Immunohistochemistry (IHC)

Immunohistochemical analysis of LITAF protein expression was performed on 50 formalin-fixed, paraffin-embedded tumor tissues, and paired adjacent non-tumor tissues. The sections were deparaffinized in xylene and rehydrated by transfer through graded concentrations of ethanol to distilled water, then placed in 500 ml of 10 mM citrate buffer (pH 6.0) and boiled for 1∼2 min. Endogenous peroxidase activity was blocked by incubation with 3% H_2_O_2_ for 10 minutes as room temperature. Sections were then treated with sheep serum to avoid non-specific staining. All sections were incubated with anti-LITAF mouse monoclonal antibody (D-5, 1:1000, Santa Cruz), overnight at 4°C, and then incubated with rabbit anti-mouse secondary antibody for 10 minutes at room temperature. After rinsing three times in PBS for 3 minutes each, the sections were incubated with DAB for 2 minutes, counterstained with hematoxylin for 2 minutes, dehydrated with gradient alcohol and transparentized with dimethylbenzene. IHC expression of LITAF was examined via light microscopy. The intensity of the IHC staining was estimated by the immunoreactive score system (IRS) (Supplementary Table 3) [28]. The IRS ≤ 1 was defined as low expression and the IRS ≥ 9 was defined as high expression. All slides were reviewed independently by 2 pathologist who were blinded to each other’s readings.

### Real-time quantitative PCR analyses

Total RNA was extracted from pancreatic cancer cells and tissues using RNAiso reagent (TaKaRa, Japan), and reverse-transcribed using a PrimeScript^®^ RT reagent kit (RR037A, Takara). Real-time PCR was performed with SYBR^®^ Premix Ex TaqTM (Takara), using the 7500 Real-Time PCR System (Applied Biosystems). The real-time primer sequences are listed in Supporting Information (Supplementary Table 4). GAPDH was used as a control. Relative gene fold change was normalized to GAPDH, and calculated using the 2^-ΔΔct^ method.

### 5-Aza-2′-deoxycytidine treatment

For 5-Aza -2′-deoxycytidine(Sigma) treatment, cell lines were treated with 1 μM，2.5 μM, or 5 μM 5-Aza-2′-deoxycytidine or an equivalent volume of dimethyl sulfoxide, for 72 h.

### Methylation-specific PCR and bisulfate genomic sequencing of *LITAF* promoter

Methylation-specific PCR (MSP) primers were designed in the 5′ untranslated region CpG island of the published sequences near translation start site of *LITAF*, as described [29]. Genbank accession: NC 018927 Region: 11680019–11680258 [30], which covers a 240bp DNA fragment harboring 16 CpG island sites.

The bisulfite modification of purified genomic DNA from four pancreatic cancer cell lines and 25 paired fresh tumor tissues was performed by using an EZ DNA Methylation Kit (ZYMO Research D5001, USA). Subsequently, MSP was carried out focusing on the target frame of *LITAF* promoter, and confirmed by direct bisulfate genomic sequencing PCR (BSP).

For BSP, bisulfite-treated DNA was amplified with primers specific for a fragment of the *LITAF* promoter that contained 16 CpG sites. The PCR products were subcloned into the pMD^™^ 19-Vector (TaKaRa, Japan) and 10–15 colonies were randomly chosen and sequenced. MSP and BSP primer sequences are listed in Supplementary Table 4.

### Proliferation assay

Cell proliferation activity was assessed with the 3-(4, 5-dimethylthiazol-2-yl)-2, 5-diphenyl tetrazolium bromide (MTT) assay. Pancreatic cells were seeded into 96-well plates at a density of 2 × 10^3^ cells/well, then the proliferation rates were measured at 0, 24, 48, 72 , 96, and 120 hours. Absorbance values were measured at 570 nm with a microplate reader. BxPC-3 and PANC-1 cells were treated with 5-Aza -2′-deoxycytidine at a concentration of 0 μM or 5 μM.

### Flow cytometry

BxPC-3 and PANC-1 cells were grown into 6-well plates at a density of 5 × 10^5^ cells/well and treated with 5-Aza -2′-deoxycytidine at a concentration of 0 μM or 5 μM for 72 h. Cells were then quantified by flow cytometry using the Annexin V/PI Apoptosis Kit (Multiscience, China). Briefly, cells were washed in cold PBS, re-suspended in 1X binding buffer, and incubated with 5 μl of FITC Annexin V and 10 μl of propidium iodide (PI) for 15 minutes in the dark. The cells were then re-suspended in 400 μl of 1X binding buffer and analyzed immediately by flow cytometry (Beckman Coulter). For cell cycle analysis, the cells were fixed in 70% ethanol and stained with 10 μl Reagent A (Multiscience, China). Then, the cells were sorted by flow cytometry (Beckman Coulter) and cell cycle profiles were analyzed by ModFit software.

### Statistical analysis

RT-qPCR, cell proliferation assay, and analysis of *LITAF* promoter methylation status were evaluated using Student’s *t*-tests. Chi-square test was used for analysis of patient clinical characteristics. The Kaplan–Meier survival analysis was used to estimate disease-free survival (DFS) and overall survival (OS). Patients were separated into two groups according to high or low expression levels of *LITAF*. The log-rank test was used to compare the survival difference between the two groups. Correlation between the *LITAF* expression and *LITAF* promoter methylation status was evaluated by calculating a Pearson correlation.

## SUPPLEMENTARY MATERIALS FIGURE AND TABLES


