# Supplementary material for: LITAF is a potential tumor suppressor in pancreatic cancer
Source: Oncotarget. 2017 Dec 14;9(3):3131–42. doi: 10.18632/oncotarget.23220 (PMC5790452; doi:10.18632/oncotarget.23220)
Supplement: Supplementary file 1 [file oncotarget-09-3131-s001.pdf]

LITAF is a potential tumor suppressor in pancreatic cancer

SUPPLEMENTARY MATERIALS

Supplementary Table 1: Clinicopathologic characteristics of patients included in this study (Only adenocarcinoma)

Characteristic	Case (%)	Median (Range)
Age(years)		
≥65	20 (50.0)	65.5 (48,82)
<65	20 (50.0)	
Gender		
male	23 (57.5)	
female	17 (42.5)	
Tumor Size(cm)		
≤2	6 (15.0)	
>2	34 (85.0)	
Lymph node involvement		
positive	22 (55.0)	
negative	18 (45.0)	
TNM Stage (WHO Classification)		
Ia	1 (2.5)	
Ib	9 (22.5)	
IIa	7 (17.5)	
IIb	20 (50.0)	
III/IV	3 (7.5)	

Supplementary Table 2: Correlation between the clinical characteristics and *LITAF* expression (Only adenocarcinoma)

	LITAF mRNA Expression (Q-PCR)				LITAF protein Expression (IHC)			
	**High**	**Low**	***N***	***p***	**High**	**Low**	***N***	***p***
Age (years)								
≥65	0	8	20	0.055	12	8	40	0.749
<65	5	7			11	9		
Gender								
male	4	6	20	0.303	13	10	40	0.884
female	1	9			10	7		
Tumor Size (cm)								
≤2	0	1	20	1.0	3	3	40	1.0
>2	5	14			20	14		
Lymph Nodes								
positive	1	10	20	0.127	12	10	40	0.676
negative	4	5			11	7		
TNM Stage								
I/II	3	15	20	0.053	21	16	40	1.0
III/IV	2	0			2	1		

Supplementary Table 3: The IRS system*

% of PPs	SI value	IRS (PP × SI)
0 = no PPs	0 = no color reaction	0–1 = negative
1 ≤ 10% PPs	1 = mild reaction	2–3 = mild
2 = 10%–50% PPs	2 = moderate reaction	4–8 = moderate
3 = 51%–80% PPs	3 = intense reaction	9–12 = strongly positive
4 ≥ 80% PPs		

*Data are according to Mehmet KT, Benjamin E, Alexander B, *et al.* [26]. IRS points 0 and 1 indicate negative expression; 2–3, positive, weak expression; 4–8, positive, moderate expression and 9–12, positive, strong expression.
Abbreviations: PP, positive cell; SI, staining intensity.

Supplementary Table 4: Sequences of the primers used in this study

Name	Primer Sequence (5′-3′)
RT-qPCR:	
LITAF F	TCGGTTCCAGGACCTTACCA
LITAF R	ACGAAGGAGGATTCATGCCC
GAPDH F	CAACGGATTTGGTCGTATTGG
GAPDH R	CTGGAAGATGGTGATGGGATT
MSP:	
LITAF M1	AAGATTCGGGAAGGATATTCG
LITAF M2	GACGATAAACGATAAACGCGTC
LITAF U1	GTAAGATTTGGGAAGGATATTTGA
LITAF U2	CCAACAATAAACAATAAACAACATC
BSP:	
LITAF F	GTAAGATTYGGGAAGGAT
LITAF R	CTTCTACCACCAATCACC

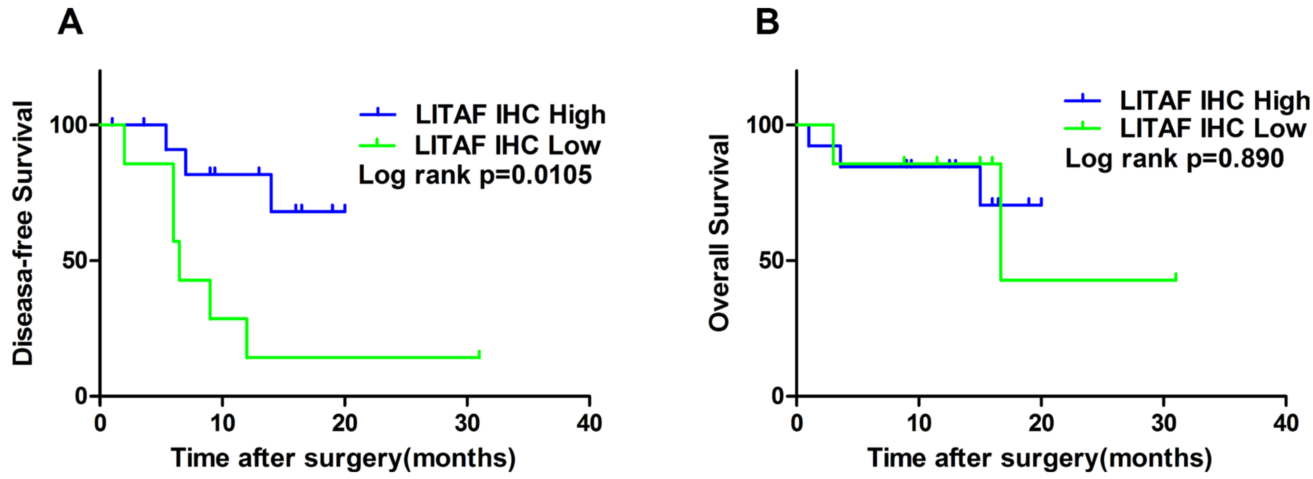

Supplementary Figure 1: LITAF protein expression correlates with survival in patients with pancreatic adenocarcinoma. The Kaplan-Meier (KM) method was used to estimate DFS and OS of the patients with pancreatic adenocarcinoma according to the *LITAF* protein expression. (**A**) The Kaplan-Meier curve for DFS revealed a poorer DFS in patients with low *LITAF* expression (median 6.5 months vs 13 months, Log rank $p = 0.0105$). (**B**) The Kaplan-Meier curve for OS showed that there was no significant difference in OS according to *LITAF* expression (median 15 vs 13 months, Log rank $p = 0.89$). Green line, Low *LITAF* expression. Blue line, High *LITAF* expression.
